# EQD2 Analyses of Vaginal Complications in Exclusive Brachytherapy for Postoperative Endometrial Carcinoma

**DOI:** 10.3390/cancers12103059

**Published:** 2020-10-20

**Authors:** Yaowen Zhang, Balbino Fornes, Gabriela Gómez, Irene Bentoldrà, Clara Carmona, Antonio Herreros, Sebastià Sabater, Inmaculada Nicolás, Yan Li, Joan Sánchez, Albert Biete, Aureli Torné, Carlos Ascaso, Ángeles Rovirosa

**Affiliations:** 1 Fonaments Clinics Department, University of Barcelona, 08036 Barcelona, Spain; yzhang@clinic.cat (Y.Z.); Fornes@clinic.cat (B.F.); ibentobo7alumnes@ub.edu (I.B.); clcarmoi7alumnes@ub.edu (C.C.); herreros@clinic.cat (A.H.); yanli@clinic.cat (Y.L.); abiete@ub.edu (A.B.); carlosascaso@ub.edu (C.A.); 2 Radiation Oncology Department, Hospital Clínic Universitari, 08036 Barcelona, Spain; 3 Radiation Oncology Department, Hospital Ángeles Chihuahua, 31217 Chihuahua, Mexico; gahumada@angeleschihuahua.com; 4 Radiation Oncology Department, Hospital General Universitario de Albacete, 02006 Albacete, Spain; ssabater@sescam.jccm.es; 5 Gynecologic Cancer Unit, Hospital Clínic Unversitari, 08036 Barcelona, Spain; Inicolas@clinic.cat (I.N.); atorne@clinic.cat (A.T.); 6 Economics Department, Hospital Clínic Universitari, 08036 Barcelona, Spain; jspérez@clinic.cat

**Keywords:** endometrial carcinoma, vaginal toxicity, vaginal dose, brachytherapy

## Abstract

**Simple Summary:**

Vaginal complications in exclusive vaginal-cuff brachytherapy (EVCBT) have not been analyzed in terms of the dose received by a vaginal volume of 2 cm^3^. The aim of this work was to analyze the vaginal dose and complications in EVCBT. In the present analysis, we found that all the patients receiving < 68Gy equivalent dose 2Gy/day at 2 cm^3^ of the most exposed area to the dose of the vagina only developed G0–G1 and 1G2 without vaginal stenosis complications and without relapses. This dose limit could eliminate G2 vaginal stenosis and is the hypothesis of a new analysis in our center.

**Abstract:**

*Background:* To evaluate whether EQD2_(α/β = 3Gy)_ at 2 cm^3^ of the most exposed area of the vagina is related to late vaginal toxicity in postoperative endometrial cancer (PEC) patients (p) treated with exclusive brachytherapy (BT). *Methods:* From 2014 to 2017, 43p were included in this study. BT was administered: 3-fractions of 6Gy in 37p and 2-fractions of 7.5Gy in 6p. The dose was prescribed at a depth of 5 mm from the applicator surface with dose-point optimization based on distance. The active treatment length was 2.5 cm. CTV-D90 and the dose to the most exposed 2 cm^3^ of the vagina was calculated for each patient. Late toxicity of the bladder and rectum was assessed using Radiation Therapy Oncology Group (RTOG) criteria, and vaginal toxicity by objective Late Effects Normal Tissue Task Force (LENT)-Subjective, Objective, Management, Analytic (SOMA) (LENT-SOMA) criteria. Statistics: frequency tables, mean, median, range, standard deviation, and box plot. *Results:* The median follow-up was 51 months (12–68). 20 p (46.5%) and 2 p (4.7%) developed G1 and G2 vaginal complications, respectively. Only 1/2 p-G2 receiving EQD2_(α/β = 3Gy)_ at 2 cm^3^ >68Gy presented vaginal shortening and 18/20 p-G1 received doses < 68Gy. *Conclusions:* PECp receiving exclusive brachytherapy with doses < 68Gy EQD2_(α/β = 3Gy)_ at 2 cm^2^ of the vagina presented only G0–G1 vaginal toxicity, except for one with bleeding telangiectasias. Larger prospective studies are necessary to confirm the present results.

## 1. Introduction

Endometrial cancer (EC) is a frequent malignant tumor of the female reproductive system with a rising incidence rate [1]. According to data from the International Agency for Research on Cancer, there were more than 380,000 new cases of EC in 2018, with the highest incidences being reported in North America, central and eastern Europe [2]. Fortunately, most cases are diagnosed by advanced imaging techniques in the early stages, with postmenopausal metrorrhagia being the most common clinical sign.

Based on clinical evidence, adjuvant brachytherapy (BT) is widely considered the standard treatment to decrease vaginal recurrence in patients with intermediate-risk EC after surgery [3,4]. However, it is difficult to compare the effectiveness of BT with that of external beam radiation (EBRT) in these patients because of the lack of consensus on BT regimens, the active length, and the dose prescription point, among other aspects. Radiation oncologists administer different schedules according to their experience and resources, with few differences in the results of vaginal-cuff relapses. Moreover, there are no in-depth studies on the effects of vaginal toxicity on the quality of life of patients with EC. One of the long-term side effects after irradiation is vaginal stenosis defined as vaginal narrowing and shortening. Vaginal stenosis can have adverse effects on sexual functioning, making gynecological examination difficult and sometimes painful, and may even impede cytological studies from detecting cancer recurrence [5]. The few studies in this respect indicate that the use of a vaginal dilator can prevent vaginal strictures. Nonetheless, according to several studies, the rate of patient compliance is generally low. The current situation highlights the need for the determination of a prognostic factor to predict vaginal toxicity.

Vaginal dose is a strong risk factor for vaginal toxicity. The reported rate of vaginal shortening or stenosis after vaginal-cuff BT is 1.6–54.7% [6,7,8,9,10]. To minimize the risk of vaginal toxicity, our center has performed 2 studies since 2018 [11,12]. In the first study, the goal was to analyze the relationship between the equivalent dose in 2Gy fractions (EQD2)_(α/β = 3Gy)_ at 0.1 cm^3^, 1 cm^3^, and 2 cm^3^ of the most exposed area of the vagina and late vaginal toxicity in 67 patients treated with BT ± EBRT after postoperative EC. It was found that 20% of the patients receiving doses higher than 68Gy (EQD2)_(α/β = 3Gy)_ at 2 cm^3^ of the most exposed area of the vagina presented G2 vaginal toxicity. In a subsequent study including 62 patients treated with EBRT+BT, it was concluded that 68Gy EQD2_(α/β = 3Gy)_ at the 2 cm^2^ of the most exposed area of the vagina could be considered as a good dose limit to minimize late G2 vaginal toxicity taking into account that 20% of the patients with a dose greater than or equal to this value presented G2 toxicity. The present study aims to analyze these results in 43 patients receiving exclusive BT.

## 2. Results

The median and mean follow-up of the patients was 51 months and 50.9 months, respectively (12–68, SD 11.2). Possible factors associated with vaginal toxicity in the literature are described in Table 1.

No vaginal-cuff recurrence or pelvic or distant metastasis were found in the 43 patients, and all the patients were alive at the time of the present analysis.

Table 2 shows the values for CTV size, D90 per fraction, CTV coverage, and EQD2_(α/β = 3Gy)_ at 2 cm^3^ of the most exposed area of the vagina and organs at risk. The difference from 49.2 to 113.4Gy could be related to the thickness of vagina, so that the lesser the thickness the higher the dose to the 2 cm^3^ of vagina, and therefore, the higher the risk of complications.

No patient presented late rectal or bladder toxicity. With regard to late vaginal toxicity, 21 patients (48.8%) did not develop any vaginal toxicity, and 20 (46.5%) and 2 patients (4.7%) developed G1 and G2 complications, respectively. Among the patients with G1 toxicity, telangiectasia was presented in 14 patients (32.6%), 4 patients (9.3%) showed <1/3 shortening in vaginal length (4 small vaginal dog ear and one patient had vaginal adherence) and both were observed in 2 patients (4.7%). Only 2 patients (4.7%) developed G2 vaginal toxicity, one as bleeding telangiectasias at examination, and the other with 1/3–2/3 shortening in vaginal length. Overall, only 7 patients presented vaginal shortening (6G1 (14.0%), and 1G2 (2.3%)) appeared at 19, 24, 34, 37, 40, 51, and 65 months, with a median time of appearance of 37 months.

**Figure 1:**
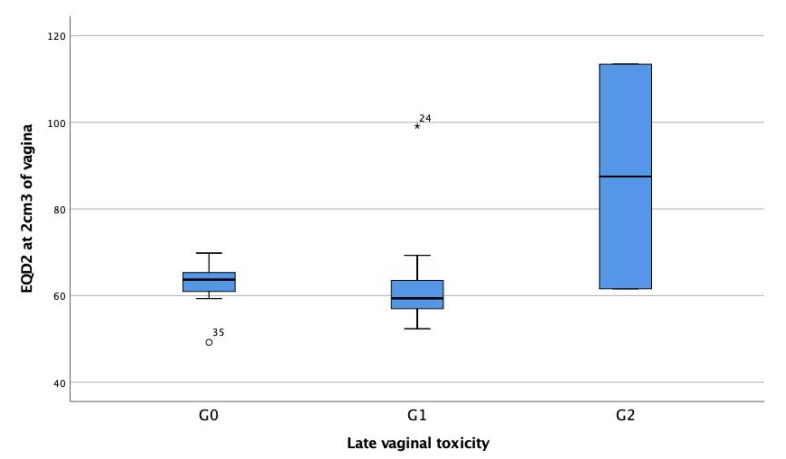
The distribution of EQD2_(α/β = 3Gy)_ in Gy at the most exposed 2 cm^3^ of vagina in relation to the grades of late vaginal toxicity.

The distribution of EQD2_(α/β = 3Gy)_ at the most exposed 2 cm^3^ of vagina in relation to the grades of late vaginal toxicity is shown in Figure 1. The mean and median EQD2_(α/β = 3Gy)_ at the most exposed 2 cm^3^ of vagina was 62.7Gy and 63.7Gy (49.2–69.8, SD 4.1) in G0 patients and 61.5Gy and 59.4Gy (52.3–99.1, SD 9.8) in G1 patients, while the dose in the 2 patients with G2 toxicity was 61.6Gy and 113.4Gy. No patient with a cylinder diameter less than 3.5 cm developed G2 complications. One of the G2 patients was treated with colpostats (113Gy at 2 cm^3^ of vagina). 

The EQD2_(α/β = 3Gy)_ dose at 2 cm^3^ in the patient with bleeding telangiectasias was 61.6Gy (cylinder diameter was 3.5 cm) and 113.4Gy (ovoid technique) in the patient with >1/3 shortening of the vagina. In the latter patient, the vagina was already short prior to treatment. The mean and median EQD2_(α/β = 3Gy)_ in patients with G0–G1 were 62.1Gy and 61.3Gy (49.2–99.1, SD 7.4). Eighteen of 20 patients with G1 toxicity received doses less than 68Gy, and 50%, and 25% of the patients receiving doses higher than 68Gy developed G1 and G2 toxicity, respectively. Dilator compliance was achieved in 20% of the patients for a period of 2 to 5 years, while 80% of the patients did not use dilators or their compliance was low during a period of less than 9 months.

## 3. Discussion

Brachytherapy is currently regarded as the recommended treatment in the management of intermediate-risk EC after surgery, mainly in patients over 60 years of age. As reported in many randomized trials, vaginal BT is effective in reducing the risk of vaginal-cuff recurrence with a low risk of complications [13,14]. The HDR BT technique has been increasingly used worldwide taking into account that it can be performed on an outpatient basis and the benefits in costs and patient comfort [15].

Brachytherapy is administered with the applicator in close contact with the vaginal mucosa, which may produce chronic radiation-induced changes including loss of capillaries, impaired microcirculation, pathological dilation of capillaries, and increased collagen production in the submucosal fibro-connective tissue layer. These alterations result in atrophy of vaginal mucosa, telangiectasias, adhesions, and occlusion of the vagina, and on rare occasions, the appearance of ulceration, necrosis, and fistulae [5,16]. As a late complication, vaginal stenosis can lead to discomfort during sexual intercourse due to dyspareunia, post-coital bleeding, and stress, resulting in sexual dysfunction which can have an adverse impact on the patient’s quality of life. It is also important to maintain the vaginal canal open in order to facilitate pap-smear screening and gynecological examination to detect tumor recurrence.

Taking into account that the vaginal mucosa in an irradiated field is less likely to heal, surgical management of vaginal stenosis is challenging. In severe cases, reconstructive surgery can sometimes be recommended based on the needs of every patient. Nonsurgical management including topical vaginal estrogen therapy may be beneficial in selected patients [17,18]. 

Vaginal dilators are used for the prevention of vaginal stenosis. A study by Bahng et al. retrospectively analyzed the medical records of 100 patients with postoperative EC who underwent adjuvant intravaginal BT between 1995 and 2009 and found that the use of a vaginal dilator at least two to three times a week was significantly associated with a decreased risk of vaginal stenosis [19]. With regard to the optimal duration of vaginal dilator use, 243 stage IA-II EC patients treated with adjuvant BT were instructed to use a vaginal dilator three times per week for at least 1 year. The results showed that extended compliance beyond 1 year was a significant predictor of reduced risk of vaginal stenosis [20]. Nevertheless, clinical evidence of the benefits of the use of vaginal dilators is still insufficient, and psychological damage and rectovaginal fistulae linked to dilator use, albeit infrequent, have been reported [21]. With the aim of analyzing the effects of a vaginal dilator on vaginal stenosis, one study included 56 consecutive patients instructed to use a vaginal dilator after completion of radiotherapy. The authors reported that vaginal dilator use had no influence on vaginal dilator diameter (*p =* 0.81), indicating that the use of vaginal dilators does not prevent vaginal stenosis or sexual impairment [22]. In the present series, only 20% of patients showed compliance with vaginal dilator use for more than 2 years, which may help to reduce the rate of G2 toxicity. Nevertheless, the small number of patients included in the present study precludes the drawing of any conclusions on the possible benefits of vaginal dilators.

As mentioned above, the prevention of vaginal stenosis should be focused on predictors of this complication. In the literature, the proportion of vagina treated > 60% (*p =* 0.009) and total dose > 14Gy (*p* = 0.015) were found to be independent predictors of Grade ≥ 1 vaginal stenosis [23]. Nevertheless, vaginal complications are associated with a greater number of factors. Banhg et al. described that older age and a shorter active length was related to a reduced risk of vaginal stenosis [19]. On the other hand, Brand et al. reported that age > 50 years was a prognostic factor associated with increased risk of vaginal toxicity (*p =* 0.02) [24]. It has also been reported that concurrent chemotherapy treatment did not increase the rate of vaginal stricture in comparison with patients treated with BT alone [25]). In the present series, the distribution of patients by age did not allow any conclusions to be drawn, and in the present study, no patient received chemotherapy. In a study including 304 EC patients treated with BT, there was a significant association between cylinder diameter and vaginal stenosis (10). In a recently published study, an increased stenosis rate was associated with a deeper prescription point (*p* = 0.005) and longer treatment length (*p* = 0.01) [26]. Since 2003 the patients in our hospital have been treated with a larger cylinder diameter, usually of 3.5 m and with a treatment source length of 2.5 cm, and patients are strongly encouraged to use vaginal dilators which could explain the low rate of G2 complications.

The topic of dose-volume histogram analysis EC BT planning evaluation is both under-studied and timely as many groups have incorporated 3-D data into their treatment planning. Contrary to analyzing the dose on the surface, it is more realistic to work with treated volumes as in other organs at risk. Since 2018 we have performed two studies analyzing the relationship between vaginal dose and late vaginal toxicity in BT in postoperative EC. We found that an EQD2_(α/β = 3Gy)_ at the most exposed 2 cm^3^ of vagina > 68Gy was linked to G2 vaginal toxicity in 67 patients treated with BT±EBRT, and also in patients treated with EBRT+BT (10, 11). These results suggested that EQD2_(α/β = 3Gy)_ < 68Gy could be a dose limit to reduce or eliminate the risk of G2 late vaginal toxicity, and that this dose constraint could also apply to patients treated with exclusive BT. Taking this into account, we carried out the present study and found that in the overall series 6 patients (13.9%) developed G1 vaginal shortening and two patients (4.7%) developed G2 vaginal toxicity, manifesting as bleeding telangiectasias in one patient and 1/3–2/3 shortening in vaginal length in another patient who received EQD2_(α/β = 3Gy)_ > 68Gy (113.4Gy) (2.3%). In a previous series from our center, the incidence of G2 complications was higher (15.3%) and the low incidence of G2 toxicity in the present study may be associated with the fact that the dose of 6Gy × 3 fractions or of 7.5Gy × 2 fractions is lower than the 5Gy × 4 fractions or 4Gy × 6 fractions commonly used [27]. 

In the present series with a long follow-up, the overall vaginal shortening was 16.2% (13.9% G1 and 2.3% G2). The 2G2 toxicities were: vaginal shortening in the patient who received 113Gy, and in the other patient, moderate and limited bleeding in the clinical examination due to telangiectasias which may justify the dose of 61.6Gy. In fact, vaginal bleeding by telangiectasias has not been analyzed in the literature.

The present study showed a higher number of G1 complications in comparison to our previous results, and this can be explained by the effect of the dose on G2 complications but not on G1 toxicity, with the patients not developing G2 developing G1 complications, although only 13.9% had G1 vaginal shortening. In the present study in which the patients received exclusive vaginal-cuff BT, it seems that a dose < 68Gy could be related to the absence of G2 vaginal stenosis, similar to our previous analysis, despite the patients having received EBRT.

Little is known about the relationship between dose-volume histogram parameters in EQD2_(α/β = 3Gy)_ and vaginal toxicity in EC. Susko et al. reported that a cut-off point of 108Gy D2cc was associated with all grades of late vaginal toxicity including G2 toxicity evaluated by CTCAE version 4.0 [28]. In 2016, the same group reported a significant association between G2+ vaginal toxicity rates and vaginal D2cc of 130Gy [29]. These differences in the doses and complications can be due to many factors such as variations in delineation of the vaginal wall, applicator type, applicator diameter, active source length, the evaluation score system for toxicity, and modalities of treatment, among others. Moreover, although the median CTV in the present analysis was 9 cm^3^, in a larger series different CTVs could also have an impact on the results. In a study by Mukarami et al. [30] in re-irradiated cervical cancer patients there was found a cut-off of 145Gy at 2 cm^3^ of vagina for ulceration as complication, considered as grade 3 in these patients This value is much higher in comparison to the 68Gy in the present series in which most of the complications were G1 with only one G2 vaginal stenosis in a patient receiving 113Gy at 2 cm^3^ of the part of the vagina most exposed to radiation.

We reached the same conclusion in the three studies carried out in our center using the same BT practice: EQD2_(α/β = 3Gy)_ > 68Gy at 2 cm^3^ of the most exposed volume of the vagina seems to be associated with late G2 vaginal toxicity. In 2018, we established this constraint for postoperative BT treatments in EC, and the preliminary results showed that no shortening of the vagina was observed using the constraint of 68Gy EQD2_(α/β = 3Gy)_ (unpublished data). Therefore, this dose limit could be reliable for eliminating the risk of G2 toxicity.

This present cohort differs from the others cited in that it is more modern and incorporates 3D dosimetry as a variable associated with late vaginal toxicity in exclusive vaginal-cuff BT, and it is also the first study to analyze different parameters of 3D planning. The vaginal CTV is both the target and the dose-limiting structure in the era of 3D planning. In the last ABS report for postoperative vaginal-cuff BT in 2017, 24 different schedules were described, most of which showed similar results in local control. The best fractionation schedule and dose have not been established in any prospective study [31]. The constraint of 68Gy EQD2 has been incorporated in our center for vaginal BT ± EBRT since 2017, and at present, the fractionation schedule is 7.5Gy × 2 fractions in exclusive treatment and 7Gy after EBRT. Considering that there is a reduction in the dose, the analysis of the minimum dose administered to the CTV in exclusive treatment is 7Gy × 2 fractions and 6Gy × 1 fraction after EBRT. Preliminary results have shown no vaginal relapses or shortening of the vagina with a median follow-up of 24 months (data not yet published).

The limitations of the present study include the small population size and its retrospective nature. Prospective studies with a larger population are required to prevent confounding results and bias.

## 4. Materials and Methods

This retrospective study included 43 EC patients consecutively treated with exclusive postoperative 3D-based BT in our department from June 2014 to March 2017 (23 of these patients were included in a previous analysis and now have a longer follow-up). The patients had been diagnosed with EC after endometrial biopsy. Imaging studies (computed tomography, ultrasonography, magnetic resonance imaging and/or positron emission tomography) were obtained before primary surgery based on the European Society of Gynaecological Oncology (ESGO) Guidelines [3]. Thirteen patients (30.2%) underwent laparoscopic-assisted vaginal hysterectomy and bilateral salpingo-oophorectomy (LAVH-BSO) with pelvic ± para-aortic lymphadenectomy; 13 patients (30.2%) only underwent LAVH-BSO. Abdominal hysterectomy plus bilateral salpingo-oophorectomy with pelvic and/or para-aortic lymphadenectomy was performed in 9 patients (20.9%) as well as in one patient (2.3%) in addition to omentectomy. Five patients (11.6%) underwent laparoscopic hysterectomy and bilateral salpingo-oophorectomy (HT-BSO), and in the remaining 2 patients (4.7%) abdominal HT-BSO was performed.

After pathological study, the 43 patients were classified as intermediate-risk (grade 1–2 with myometrial invasion ≥50%, grade 3 with myometrial invasion <50%, or grade 1–2 with lymphovascular space invasion regardless of the depth of myometrial invasion; tumor size >2 cm and age >60 years were also considered in the selection of patients for this treatment) and underwent exclusive vaginal BT. Table 3 shows the characteristics of the patients. The present study had institutional review board approval from the Hospital Clinic of Barcelona (HCB) with number HCB/2014/0575.

High-dose-rate (HDR) BT was administered in all the patients with 2 different daily administration schedules: 3 fractions of 6Gy in 37 patients and 2 fractions of 7.5Gy in 6 patients. The first application was carried out in the operating room, where the patients were examined to assess the healing status of the vaginal cuff and determine the type of applicator that each patient required. Cylinders were most commonly used, and the largest possible diameter was chosen to reduce the dose on the mucosal surface. Overall, for the vaginal cylinder technique, a diameter of 3.5 cm was used in 37 patients, 3 cm in 3 patients, and 2.5 cm in 1 patient. Ovoids were used in 2 patients with small introitus and a wide vaginal cuff. The BT procedure for applicator placement has been described elsewhere [32].

**Figure 2:**
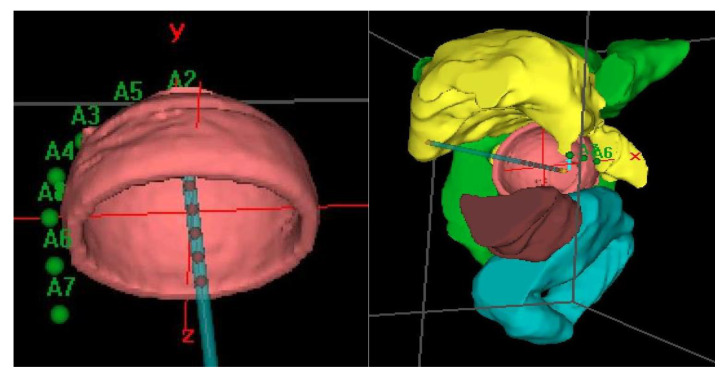
Clinical target Volume (CTV) and Organs at risk (OAR) delineation.

Prior to treatment, a computerized tomography (CT) simulation scan was performed with image acquisition every 1 mm for 3D planning. Special care was taken to confirm close contact of the applicator to the vagina in the CT in order to avoid relapses. Our procedure for clinical target volume (CTV) delineation consists of generating an automatic contour based on the difference in Hounsfield units of the applicator and the patient’s tissues. From this automatic contour, we generate an isotropic 3 mm margin, that is, the expansion volume. Then, a boolean subtraction of the applicator from the expansion volume is made. Finally, manual delineation of the external part of this volume is performed to adapt it to the real thickness of the vagina, which can vary depending on the case and cylinder diameter. For manual delineation, a 2 mm diameter pearl tool is used. The CTV was delineated along the first cylinder and the mean length of the postoperative treated vaginal cuff was 3cm; the rectum, bladder, and sigma were also delineated along 2 cm from the top to 2 cm under the CTV using the Oncentra Brachy planning system (V.4.5.3) (Elekta^®^,Nucletron BV, Veenendaal, The Netherlands). (Figure 2). 

**Figure 3:**
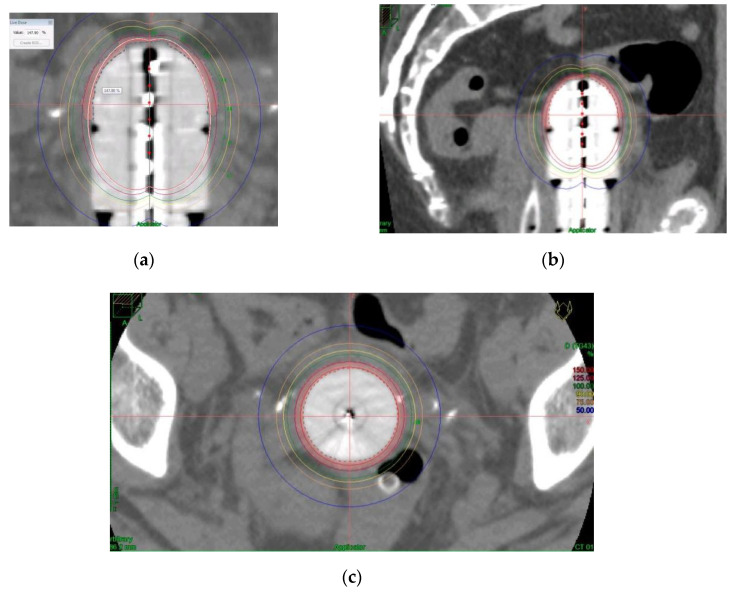

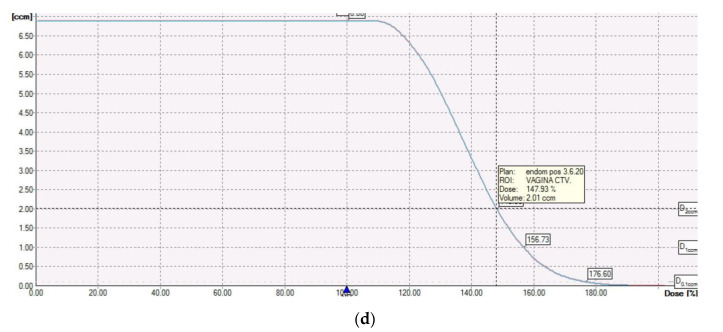
Example of dosimetric study showing the most exposed 2 cm^3^ of vagina to the dose in coronal (**a**), sagittal (**b**) and axial (**c**) planes and (**d**) dose volume histogram.

The dose was prescribed at a depth of 5 mm from the applicator surface, and point dose optimization based on distance was used. The active treatment length was 2.5 cm. In the present study, 90% of the isodose surface included the CTV. The voxels corresponding to the vaginal D2cc are always located in a small volume at the top of the vaginal-cuff and any reduction in the dose at this level will consequently reduce the dose at the prescription point. This 2cc volume is described and presented in Figure 3.

Distension of the rectum and air gaps have no impact on the D2cc of vagina considering that the 90% isodose always includes the CTV. The minimum dose of 90% received by 90% of the hottest CTV volume (D90) and the percentage of CTV coverage were calculated as well as the dose to the most exposed 2 cm^3^ of the rectum, bladder, and vagina. An EQD2_(α/β = 3Gy)_ constraint of 65Gy for rectum and 80Gy for bladder were applied but were never necessary. Dose distribution was not modified to exclude the organs at risk from the dose prescription area. Figure 2 shows the dosimetric study with the area of the vagina most exposed to the dose. After dosimetric evaluation patients underwent treatment with an HDR microSelectron v2 Iridium 192 source and an afterloading source projector (Nucletron^®^ microSelectron V3 digital ELEKTA, Holland, The Netherlands).

The patients were followed for 15 days after the end of treatment and then every 3–4 months during the first 2 years followed by every 6 months up to 5 years. According to the protocol of the Gynecological Cancer Unit, at each visit, the patients were evaluated by clinical examination and radiological methods for the presence of vaginal, pelvic, or distant recurrence. The patients were evaluated by the same expert in the Radiation Oncology Department. The presence of complications was determined by gynecological examination, clinical interview, and other tests if necessary. Considering the age of the study population sexual activity was not included in the analysis, and the patients were recommended to use dilators for the prevention of vaginal stenosis during the 5-year follow-up.

Factors associated with vaginal toxicity (age, BMI, use of vaginal dilators, cylinder diameter, CTV and EQD2 to the most exposed area of the vagina) in the literature were recorded.

Late toxicity of bladder and rectum was assessed using RTOG criteria, and vaginal toxicity by objective LENT-SOMA criteria [33,34]. Vagina Grade 1 was defined as >2/3 normal vaginal length, superficial ulceration (≤1cm^2^), patchy atrophy, and telangiectasia without bleeding. Grade 2 was defined as 1/3–2/3 normal length, symptomatic dryness, superficial ulceration (>1cm^2^), confluent atrophy, telangiectasia with gross bleeding and partial synechiae. Grade 3 was defined as <1/3 normal vaginal length, dysfunction secondary to dryness, deep ulceration, nonconfluent atrophy, complete synechiae and intermittent bleeding, and Grade 4 vaginal toxicity was defined as obliteration, fistulae, diffuse atrophy, and persistent bleeding.

### Statistical Analysis

Description of the qualitative variables was performed using frequency tables and calculating the mean, median, range, and standard deviation for the quantitative variables. The equivalent dose in 2Gy fractions (EQD2) with an α/β of 3Gy to the most exposed 2 cm^3^ volume of vagina was calculated by a formula of n·d·(d+3)/5 where n is the number of fractions, and d, the dose to the most exposed 2 cm^3^ of vagina. The distribution of EQD2_(α/β = 3Gy)_ at the most exposed 2 cm^3^ of vagina in relation to the grades of late vaginal toxicity was expressed in a Box Plot. All the above analyses were performed using the statistical package SPSS v.25.

## 5. Conclusions

In the present retrospective analysis, patients who received exclusive BT for postoperative EC with doses less than 68Gy EQD2_(α/β = 3Gy)_ at 2 cm^2^ of the most exposed volume of the vagina presented only G0-G1 vaginal complications, with the exception of one patient who presented bleeding telangiectasias. Prospective studies with a larger number of patients are needed to confirm the present results.

## Figures and Tables

**Table 1:** Shows the distribution of the possible factors related to vaginal toxicity in the present series.

Possible Factors Associated with Vaginal Toxicity	Number/%
Age (years): <50	2 (4.7%)
50–55	4 (9.3%)
56–60	6 (14.0%)
>60	31 (72.1%)
Body mass index: <35	17 (39.5%)
≥35	7 (16.3%)
Not available	19 (44.2%)
Cylinder diameter (cm): <3.5	4 (9.3%)
≥3.5	37 (86.0%)
Colpostats	2 (4.7%)
CTV size (cm^3^): <9 cm^2^	22 (51.2%)
≥9 cm^2^	21 (48.8%)
Dilator compliance: None or less 9 months	28 (65.1%)
≥2 years	15 (34.9%)
EQD2 D2 cm^3^ Vagina (α/β = 3): <68Gy	39 (90.7%)
≥68Gy	4 (9.3%)

CTV: Clinical target volume.

**Table 2:** Shows the D90, the CTV size in cm^3^, the coverage of CTV, and EQD2_(α/β = 3Gy)_ at the most exposed 2 cm^3^ of the vagina, bladder, and rectum.

Value Parameters of the CTV and OAR	CTV Size (cm^3^)	D90 per Fraction (Gy)	CTV Coverage (%)	Vaginal D2 cm^3^ (Gy)	Rectum D2 cm^3^ (Gy)	Bladder D2 cm^3^ (Gy)
Mean	9.1	7.0	99.5	63.3	23.1	22.0
Range	6.5–14.0	5.5–9.0	91.2–100	49.2–113.4	10.2–42.0	11.0–31.3
Median	9.0	6.9	100	61.6	22.2	22.5
SD	1.77	0.75	1.87	10.6	6.30	4.96

SD: standard deviation, CTV: Clinical Target Volume, OAR: Organs at Risk.

**Table 3:** Pathological characteristics of the patients.

Mean Age (years)	64.6 (47–79, SD 8.2)
2009 FIGO stage	
IA	29 (67.4%)
IB	14 (32.6%)
Histological type	
Endometrioid	42 (97.7%)
Mixed	1 (2.3%)
Grade	
1	14 (32.6%)
2	24 (55.8%
3	5 (11.6%)
Myometrial invasion	
No	1 (2.3%)
≤50%	28 (65.1%)
>50%	14 (32.6%)
VLSI	
No	41 (95.3%)
Yes	1 (2.3%)
NA	1 (2.3%)
Mean tumor size (cm)	3.2(1–7, SD 1.2)

VLSI: vascular lymphatic space invasion, NA: not available, SD: standard deviation.

## References

[1] Lortet-Tieulent J., Ferlay J., Bray F., Jemal A. (2018). International Patterns and Trends in Endometrial Cancer Incidence, 1978–2013. J. Natl. Cancer Inst..

[2] Corpus Uteri. https://gco.iarc.fr/today/data/factsheets/cancers/24-Corpus-uteri-fact-sheet.pdf.

[3] Colombo N., Creutzberg C., Amant F., Bosse T., González-Martín A., Ledermann J., Marth C., Nout R., Querleu D., Mirza M.R. (2016). ESMO-ESGO-ESTRO Consensus Conference on Endometrial Cancer: Diagnosis, treatment and follow-up. Ann. Oncol..

[4] Sabater S., Andres I., Lopez-Honrubia V., Berenguer B., Sevillano M., Jimenez-Jimenez E., Rovirosa A., Arenas M. (2017). Vaginal cuff brachytherapy in endometrial cancer—A technically easy treatment?. Cancer Manag. Res..

[5] Morris L., Do V., Chard J., Brand A.H. (2017). Radiation-induced vaginal stenosis: Current perspectives. Int. J. Women’s Health.

[6] Noyes W.R., Bastin K., Edwards S.A., Buchler D.A., Stitt J.A., Thomadsen B.R., Fowler J.F., Kinsella T.J. (1995). Postoperative vaginal cuff irradiation using high dose rate remote afterloading: A phase II clinical protocol. Int. J. Radiat. Oncol. Biol. Phys..

[7] Nunns D., Williamson K., Swaney L., Davy M. (2000). The morbidity of surgery and adjuvant radiotherapy in the management of endometrial carcinoma. Int. J. Gynecol. Cancer.

[8] Onsrud M., Strickert T., Marthinsen A.B. (2001). Late reactions after postoperative high-dose-rate intravaginal brachytherapy for endometrial cancer: A comparison of standardized and individualized target volumes. Int. J. Radiat. Oncol. Biol. Phys..

[9] Chong I., Hoskin P.J. (2008). Vaginal vault brachytherapy as sole postoperative treatment for low-risk endometrial cancer. Brachytherapy.

[10] Qian J.M., Stahl J.M., Young M.R., Ratner E., Damast S. (2017). Impact of vaginal cylinder diameter on outcomes following brachytherapy for early stage endometrial cancer. J. Gynecol. Oncol..

[11] Del Valle Aguilera M., Rovirosa Á., Ascaso C., Herreros A., Sánchez J., Garcia-Migue J., Cortes S., Agusti E., Camacho C., Zhang Y. (2018). Late G2 vagina toxicity in post-operative endometrial carcinoma is associated with a 68 Gy dose equivalent to 2 Gy per fraction(α/β = 3Gy) at 2 cm3 of vagina. J. Contemp. Brachyther..

[12] Zhang Y., Ascaso C., Herreros A., Sánchez J., Sabater S., Del Pino M., Li Y., Gómez G., Torné A., Biete A. (2020). Postoperative endometrial carcinoma treated with external beam irradiation plus vaginal-cuff brachytherapy. Is there a dose relationship with G2 vaginal complications?. Rep. Pract. Oncol. Radiother..

[13] Wortman B.G., Creutzberg C.L., Putter H., Jürgenliemk-Schulz I.M., Jobsen J.J., Lutgens L.C.H.W., van der Steen-Banasik E.M., Mens J.W.M., Slot A., Stenfert Kroese M.C. (2018). Ten-year results of the PORTEC-2 trial for high-intermediate risk endometrial carcinoma: Improving patient selection for adjuvant therapy. Br. J. Cancer.

[14] Nout R., Smit V., Putter H., Jürgenliemk-Schulz I.M., Jobsen J.J., Lutgens L.C.H.W., van der Steen-Banasik E.M., Mens J.W.M., Slot A., Stenfert Kroese M.C. (2010). Vaginal brachytherapy versus pelvic external beam radiotherapy for patients with endometrial cancer of high-intermediate risk (PORTEC-2): An open-label, non-inferiority, randomised trial. Lancet.

[15] Nag S. (2004). High Dose Rate Brachytherapy: Its Clinical Applications and Treatment Guidelines. Technol. Cancer Res. Treat..

[16] Kirchheiner K., Fidarova E., Nout R.A., Schmid M.P., Sturdza A., Wiebe E., Kranz A., Polterauer S., Pötter R., Dörret W. (2012). Radiation-induced morphological changes in the vagina. Strahlenther. Onkol..

[17] Wolf J.K. (2006). Prevention and treatment of vaginal stenosis resulting from pelvic radiation therapy. Community Oncol..

[18] Denton A.S., Maher E.J. (2003). Interventions for the physical aspects of sexual dysfunction in women following pelvic radiotherapy. Cochrane Database Syst. Rev..

[19] Bahng A.Y., Dagan A., Bruner D.W., Lin L.L. (2012). Determination of prognostic factors for vaginal mucosal toxicity associated with intravaginal high-dose rate brachytherapy in patients with endometrial cancer. Int. J. Radiat. Oncol. Biol. Phys..

[20] Stahl J.M., Qian J.M., Tien C.J., Carlson D.J., Chen Z., Ratner E.S., Park H.S., Damast S. (2019). Extended duration of dilator use beyond 1 year may reduce vaginal stenosis after intravaginal high-dose-rate brachytherapy. Support Care Cancer.

[21] Miles T., Johnson N. (2014). Vaginal dilator therapy for women receiving pelvic radiotherapy. Cochrane Database Syst. Rev..

[22] Akbaba S., Oelmann-Avendano J.T., Krug D., Arians N., Bostel T., Hoerner-Rieber J., Nicolay N.H., Debus J., Indel K., Foerster R. (2019). The impact of vaginal dilator use on vaginal stenosis and sexual quality of life in women treated with adjuvant radiotherapy for endometrial cancer. Strahlenther. Onkol..

[23] Park H.S., Ratner E.S., Lucarelli L., Polizzi S., Higgins S.A., Damast S. (2015). Predictors of vaginal stenosis after intravaginal high-dose-rate brachytherapy for endometrial carcinoma. Brachytherapy.

[24] Brand A.H., Bull C.A., Cakir B. (2006). Vaginal stenosis in patients treated with radiotherapy for carcinoma of the cervix. Int. J. Gynecol. Cancer.

[25] Nieto K., Martin B., Pham N., Palmere L., Silva R.S., Winder A., Liotta M., Potkul R.K., Harkenrider M.M. (2018). Does adjuvant concurrent or sequential chemotherapy increase the radiation-related toxicity of vaginal brachytherapy for endometrial cancer patients?. Brachytherapy.

[26] Guy C.L., Fields E.C., Quinn B.A., Fisher C.M., Ladbury J.C., Romano K.C., Todor D. (2019). The vaginal cylinder: Misunderstood, misused, or trivial? An in-depth dosimetric and multiinstitutional outcome investigation. Brachytherapy.

[27] Rovirosa Á., Herreros A., Camacho C., Ascaso C., Sánchez J., Cortés S., Sabater S., Solà J., Torné A., Arenas A. (2017). Comparative results of three short brachytherapy schedules as exclusive treatment in postoperative endometrial carcinoma. Brachytherapy.

[28] Susko M., Craciunescu O., Meltsner S., Yang Y., Steffey B., Cai J., Chino J. (2016). Vaginal Dose Is Associated With Toxicity in Image Guided Tandem Ring or Ovoid-Based Brachytherapy. Int. J. Radiat. Oncol. Biol. Phys..

[29] Susko M., Craciunescu O., Meltsner S. (2016). Vaginal Toxicity From Vaginal Brachytherapy and Capri-Based Systems. Int. J. Radiat. Oncol. Biol. Phys..

[30] Mukarami N., Kasematsu T., Sumi M., Yoshimura R., Harada K., Kitaguchi M., Kana S., Sekii S., Takahashi T., Yoshio K. (2014). Vaginal Tolerance of CT image-guided high-dose-rate interstitial brachytherapy for gynecological cancer. Radiat. Oncol..

[31] Harkenrider M.M., Block A.M., Alektiar K.M., Gaffney D.H., Jones E., Klopp A., Viswanathan A.K., Small W. (2017). American Brachytherapy Task Group Report: Adjuvant vaginal brachytherapy for early-stage endometrial cancer: A comprehensive review. Brachytherapy.

[32] Rovirosa A., Ascaso C., Sánchez-Reyes A., Herreros A., Abellana R., Pahisa J., Lejarcegui J.A., Biete A. (2011). Three or four fractions of 4-5 Gy per week in postoperative high-dose-rate brachytherapy for endometrial carcinoma. Int. J. Radiat. Oncol. Biol. Phys..

[33] (1995). Late effects consensus conference: RTOG/EORTC. Radiother. Oncol..

[34] (1995). LENT SOMA scales for all anatomic sites. Int. J. Radiat. Oncol. Biol. Phys..

